# Complex and pleiotropic signaling pathways regulated by the secreted protein augurin

**DOI:** 10.1186/s12964-023-01090-8

**Published:** 2023-04-11

**Authors:** Margaux Richter, Enzo Lalli, Carmen Ruggiero

**Affiliations:** 1 Institut de Pharmacologie Moleculaire et Cellulaire CNRS UMR 7275, Valbonne, France; 2 Universite Cote d’Azur, Valbonne, France; 3 Inserm, Valbonne, France

**Keywords:** ECRG4, Augurin, Signal transduction, Peptides

## Abstract

The secreted protein augurin, the product of the tumor suppressor gene *Ecrg4*, has been identified as a peptide hormone in the human proteome in 2007. Since then, a number of studies have been carried out to highlight its structure and processing and its potential roles in physiopathology. Although augurin has been shown to be implicated in a variety of processes, ranging from tumorigenesis, inflammation and infection to neural stem cell proliferation, hypothalamo-pituitary adrenal axis regulation and osteoblast differentiation, the molecular mechanisms of its biological effects and the signaling pathways it regulates are still poorly characterized. Here we provide a comprehensive overview of augurin-dependent signal transduction pathways. Because of their secreted nature and the potential to be manipulated pharmacologically, augurin and its derived peptides represent attractive targets for diagnostic development and discovery of new therapeutic agents for the human diseases resulting from the deregulation of the signaling cascades they modulate. From this perspective, the characterization of the precise nature of augurin derived peptides and the identification of the receptor(s) on the cell surface conveying augurin signaling to downstream effectors are crucial to develop agonists and antagonists for this protein.

**Video abstract**

**Supplementary Information:**

The online version contains supplementary material available at 10.1186/s12964-023-01090-8.

## Background

Synchronous and regulated messaging within our body contributes to maintain physiological homeostasis. Hormones, endogenous peptides and growth factors are key actors in conveying this message flow between different cellular types and organs. An altered or improperly regulated secretion of those agents could initiate a disease condition or worsening an existing one. Peptide hormones are processed and secreted as small proteins that signal via membrane receptors and play important roles in virtually every aspect of physiology. Together with their receptors, they can be considered as important diagnostic and therapeutic targets. Indeed, as intrinsic signaling molecules for several physiological functions, peptide hormones represent an opportunity for therapeutic interventions that closely mimic physiological pathways. Augurin, the product of the tumor suppressor gene *Ecrg4*, was identified as a novel peptide hormone in the human proteome by Mirabeau and co-workers [1]. Since then, a number of studies have been carried out to highlight its precise structure, processing and potential roles in physiological and pathological processes. Although a wide number of functions in health and disease have been assigned to augurin, the mechanisms of its biological effects and the signaling pathways it regulates are still poorly characterized. Here we provide a comprehensive overview of the signaling cascades in which augurin has been shown to be involved.

## Discovery and protein processing

**Fig. 1:**
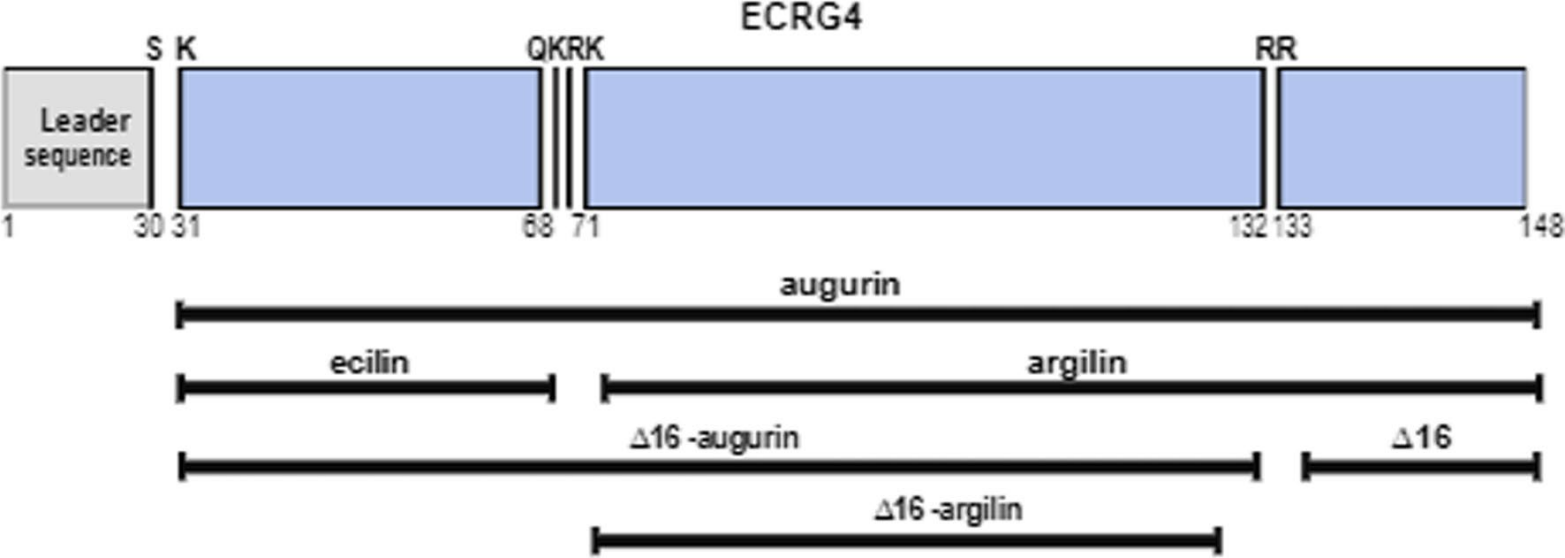
Predicted processing of ECRG4. Schematic representation of the potential protein products which have been predicted by algorithms to originate from ECRG4: intact ECRG4 (aa. 1–148), its leader sequence (aa. 1–30), augurin (aa. 31–148), ecilin (aa. 31–68), argilin (aa. 71–148) and their respective Δ16C-terminal cleaved homologs: CΔ16-augurin (aa. 31–130), CΔ16-argilin (aa. 71–130) and the Δ16 peptide itself (aa. 133–148) [3]. A putative furin-like cleavage (aa. 68–71) and a predicted thrombin cleavage site (aa. 130–134) are shown

Esophageal cancer-related gene 4 (*ECRG4)*, the gene coding for augurin*,* is highly conserved among vertebrates and is located on the chromosome 2q12.2. It was first cloned by Su and colleagues and described as a tumor suppressor in human esophageal epithelium [2]. Since this discovery, several studies have been carried out to better understand the biological functions of augurin. In 2007, Mirabeau and co-workers [1] in the attempt to identify genes encoding peptide hormones in the human genome, described one potential candidate encoded by *C2orf40*, which lies in the same locus as *ECRG4*. They were the first to show that this gene codes for a protein of 17 kDa which displays all the features of a secreted neuropeptide hormone precursor, containing a leader peptide (aa. 1–30), a putative furin-like cleavage site (aa. 68–71) and a predicted thrombin cleavage site (aa. 130–134) [3, 4]. Different potential protein products have been predicted by algorithms to be generated from the processing of the augurin precursor ECRG4 (aa. 1–148): the leader sequence (see above), augurin (aa. 31–148, originated from the cleavage of the leader peptide sequence); ecilin (aa. 31–68); argilin (aa. 71–148), ecilin and argilin being produced by the cleavage of augurin at the putative furin-like cleavage site (see above); the Δ16C-terminal cleaved homologs CΔ16-augurin (aa. 31–130) and CΔ16-argilin (aa. 71–130); the Δ16 peptide itself (aa 134–148) [1, 3, 4] (Fig. 1).

Augurin was initially shown to undergo trafficking into dense core granules of the secretory pathway [1], a common characteristic of peptide hormones. Several studies have provided evidence for the presence of ECRG4-derived peptides in normal tissue lysates, biological fluids, like the cerebrospinal one, and in the conditioned media of cells that have been transfected with an expression vector for *ECRG4* [4–9].

However, differently from traditionally secreted neuropeptide precursors, ECRG4 was shown to localize to the surface of epithelial cells and to remain tethered after secretion [4], its releasing from tethering appearing to be cell-type dependent [4]. ECRG4 thus seems not to act as a pre-pro-neuropeptide protein precursor but is rather reminiscent of a growth factor or a cytokine/chemokine, which are cell membrane-associated proteins that are released after cell surface processing [8–10].

## Expression pattern

The *ECRG4* gene is expressed in a wide range of normal human and rodent tissues like bone marrow, brain, eye, heart, intestine, kidney, lung, placenta, skeletal muscle, skin, spleen, esophagus, prostate [1, 2, 11–13]. Its expression has been identified to be the highest in adrenal glands, ovaries, retina and trachea in mice, and in thyroid, pituitary, testis and adrenal glands in humans [14].

In the mouse central nervous system (CNS) the *Ecrg4* gene and its product augurin have been predominantly localized to the choroid plexus [3]. They are also expressed in the olfactory bulb, the cerebellum, the hypothalamus and the amygdala in humans as well as in mice [14]. *Ecrg4*/augurin expression has so far been essentially reported in epithelial cells, including more specialized epithelial-derived ones, both in the CNS [pituicytes [15], oligodendrocytes [16]*,* astrocytes [6], choroid plexus epithelium [17] and ventricular ependymal cells [18, 19]] and outside the CNS [mucosal [20], epidermal [21], corneal [7], airway epithelial cells [21] and chondrocytes [5]]. Finally, *Ecrg4*/augurin expression has also been reported on the surface of circulating neutrophils and lymphocytes [22]. This wide pattern of distribution suggests that augurin has important functions, being involved in development and tissue homeostasis.

## Functions

A number of studies have been carried out with the aim of better characterizing the role of augurin in physiopathology. They have highlighted the involvement of augurin in several crucial processes at the interface of inflammation, immunity, tumorigenesis, neurobiology, and endocrinology.

Remarkably, *ECRG4* is downregulated in the vast majority of cancer types compared to the corresponding normal tissues, displaying the characteristics of a tumor suppressor gene [23]. It has been shown that the *ECRG4* promoter undergoes aberrant methylation which causes extinction of its expression in a variety of different human cancers, such as esophageal [24] and colorectal cancer [25], malignant glioma [26, 27], breast [28] and prostate cancer [29]. Indeed, the tumor suppressor function of augurin has been well described in vitro by overexpressing the protein in cancer cells, producing an increased apoptosis and a decreased proliferation rate [27, 30]. In vivo studies have also provided evidence of important antitumorigenic effects of augurin [23, 31–33].

The role of augurin in injury, inflammation and infection has been widely documented [34, 35]. Indeed, *Ecrg4* expression has been shown to decrease after CNS injury in zebrafish embryos [3], as well as after brain injury in rats [19]. A rapid decrease of *Ecrg4* expression after injury has been reported to coincide with neural progenitor cell (NPCs) proliferation, suggesting an inhibitory function for augurin in normal CNS. On the other hand, the decreased expression of *Ecrg4* after injury has been described to promote proliferation [3]. *Ecrg4* gene expression was also downregulated after infection in quiescent middle ear mucosa, concomitantly with increased cell proliferation [20]. Furthermore, increased *Ecrg4* gene expression has been observed in models of chronic inflammation [5, 20] and in skin after cutaneous burn injury [22]. Overall, those data indicate that augurin is involved in maintaining epithelium homeostasis via a potential sentinel role by coordinating the proliferative and inflammatory responses.

Augurin has also been shown to have essential functions in the CNS. A study reported an increased *Ecrg4* expression in oligodendrocyte precursor cells (OPCs) and NPCs in the aged mouse brain as well as in senescent OPCs [16]. Furthermore, augurin overexpression has been shown to promote cell-cycle arrest in OPCs and induce their senescence [16], those results suggesting that augurin is a factor linking neural cell senescence and aging. More recently, Nakatani and colleagues demonstrated recovered age-dependent decline in neural stem cells (NSC) proliferation associated with enhanced learning and memory [17] in an *Ecrg4*-deficient mouse model.

**Fig. 2:**
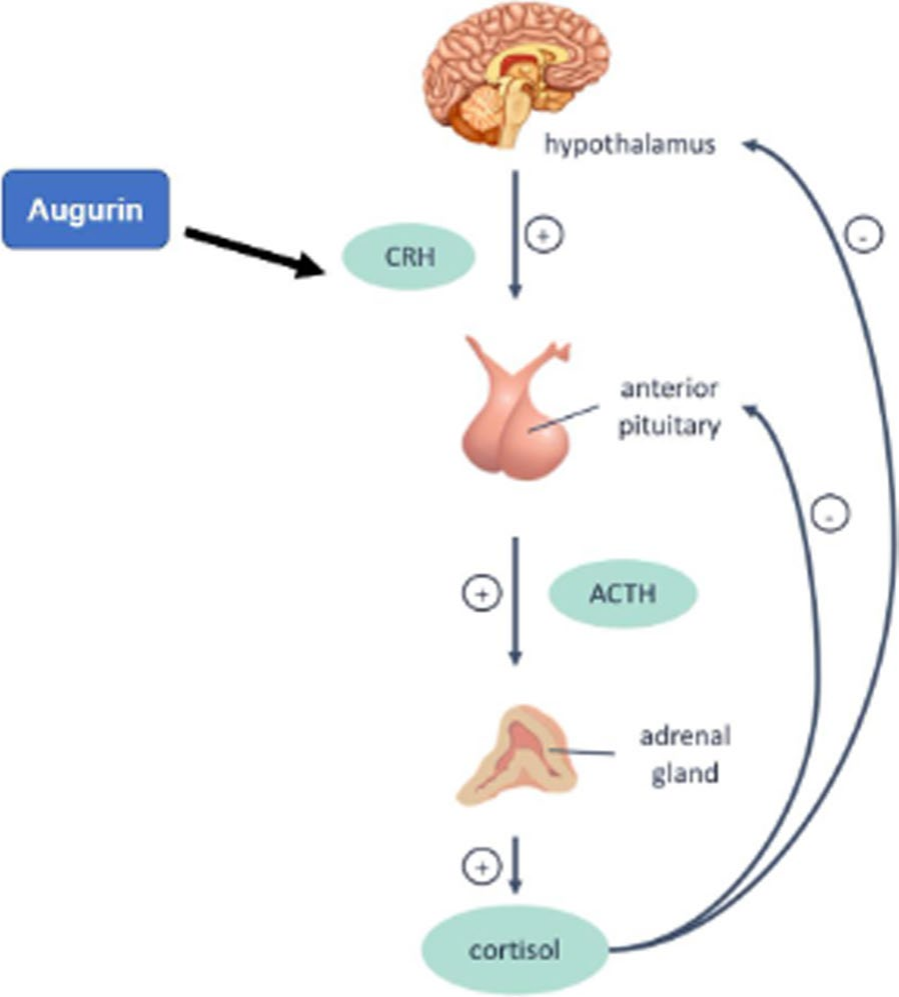
Augurin implication in the HPA axis. Upon an external or internal stressor, the paraventricular nucleus (PVN) of the hypothalamus induces the release of corticotropin releasing hormone (CRH), which in turn stimulates the release of the adrenocorticotropin hormone (ACTH) from the pituitary gland. ACTH promotes the synthesis and release of glucocorticoids (*e.g*. corticosterone in rodents and cortisol in humans) by the adrenal gland. Augurin stimulates the activation of the HPA axis via the release of CRH in rats [36]. A negative feedback response has been described involving cortisol/corticosterone—mediated rapid inhibition of HPA axis activity though inhibition of CRH and ACTH secretion from the PVN and anterior pituitary, respectively

A neuroendocrine function for augurin has also been reported [36]. Indeed, Tadross and co-workers have shown that augurin stimulates the hypothalamo-pituitary-adrenal (HPA) axis via the release of corticotropin-releasing hormone (CRH) in rats (Fig. 2).

Overall, those findings suggest that the pharmacological manipulation of the augurin system might be a new target for the modulation of the HPA axis.

We have recently shown that *Ecrg4* expression is strongly increased during the process of osteoblast differentiation in vitro and that osteoblasts from *Ecrg4* −/− mice have an accelerated differentiation, which indicates a role for augurin in osteoblast differentiation [37].

A detailed description of augurin functions is beyond the aims of the present review. However, we would like to emphasize that the molecular mechanisms underlying the biological functions of augurin remain largely unexplored.

## Signal transduction

As reported above, augurin is expressed in several tissues, and a variety of biological functions has been ascribed to it. Below we provide an overview of the signal transduction pathways it regulates. It is important to underline that for the definition of the signaling pathways modulated by augurin the experimental strategy in most of the cases has been to transfect cells to overexpress the *Ecrg4* gene and then analyze potential changes in the expression, activation/ inhibition of specific signaling molecules. The identification of naturally processed ECRG4-derived peptides being still elusive, whether the pathways described below are modulated by the full-length form of the protein or by one of the different derived peptides has not been clearly defined, unless specified, for each study presented.

### NF-κB signaling pathway

The transcription factor nuclear factor kappa-light-chain-enhancer of activated B cells (NF-κB) plays important roles in a large variety of physiological and pathological processes. NF-κB is the nuclear effector of signaling pathways responding to various extracellular stimuli like cytokines or growth factors. An activation of those signaling cascades may trigger inflammation, innate and adaptive immune responses, cell proliferation and survival [38, 39]. In mammals, the NF-κB family is composed of five related transcription factors: p50, p52, RelA (p65), c-Rel, and RelB [40]. They can bind to promoters and enhancer regions of a wide variety of target genes like those encoding cytokines and chemokines or those regulating the survival, activation and differentiation of innate immune cells and inflammatory T cells. The activation of NF-κB implicates two major signaling pathways, the canonical and noncanonical one, both being important for modulating immune and inflammatory responses despite their differences in signaling mechanisms. Multiple studies have reported an augurin involvement in the modulation of the NF-κB signaling in different cell lines.

It has been shown that the soluble augurin-derived peptide CΔ16 (aa. 133–148) can activate NF-κB signaling [22]. Indeed, the phosphorylated form of p-65 (p-p65), a marker of NF-κB activity, has been shown to increase in mouse peritoneal macrophages treated with this peptide. It has been hypothesized that both augurin and its processed form CΔ16 form a complex on innate immune cells with a candidate receptor, the Toll like receptor 4 (TLR4), activating the non-canonical NF-κB pathway [41, 42]. Another study has proposed that augurin-mediated NF-κB pathway activation relies on other receptors than TLR4, augurin having been recently described as a ligand of multiple scavenger receptors [43]. Indeed, the LOX1 scavenger receptor has been reported to facilitate augurin internalization and lead to MyD88-dependent NF-κB activation in microglia [43]. The mechanisms by which augurin activates NF-κB pathway in macrophages have not yet been elucidated, however this activation seems to be dependent on both scavenger receptors and TLRs.

Other regulatory mechanisms have been proposed in cancer cell lines. Augurin overexpression inhibited the expression of NF-κB and its nuclear translocation in esophageal squamous cell carcinoma [23]. Furthermore, a decreased expression of COX-2, which is a NF-κB target, was observed [23]. A more recent study shed light on the augurin-mediated NF-κB signaling inhibition in a panel of bladder cancer cell lines (J82, BC-5367, UMUC3, BIU-87, and T24) [44]. To confirm the implication of augurin in NF-κB signaling, the effect of *Ecrg4* expression on the NF-κB transcriptional activity has been investigated using an NF-κB luciferase reporter. Interestingly, it has been shown that *Ecrg4* overexpression decreased, whereas its knockdown increased NF-κB activity [44]. A regulatory mechanism has been described whereby augurin upregulates nuclear factor 1 C-type (NFIC), which can bind to the promoter region of osteoglycin (OGN) regulating its transcription [44]. Increased OGN causes the inhibition of NF-κB signaling in bladder cancer cells, resulting in repression of cell proliferation and invasion [44]. Those results indicate that both augurin and OGN function as tumor repressors, overexpression of augurin inhibiting the NF-κB signaling cascade via the stimulation of the NFIC/OGN signaling.

Altogether, those studies show that augurin is implicated in NF-κB signaling regulation, the outcomes of this modulation being cell type—dependent. The possibility of using augurin and derived peptides to pharmacologically modulate the NF-κB signaling cascades is promising for the control of inflammation and inflammatory diseases. However, the precise molecular mechanisms underlying augurin involvement in NF-κB signaling remain to be fully elucidated.

### PI3K/Akt/mTOR signaling pathway

The phosphatidylinositol-3-kinase (PI3K)/Akt and the mammalian target of rapamycin (mTOR) signaling cascades are two pathways critical for several aspects of cell growth and survival, in both physiological and pathological conditions. They are so interconnected that they could be considered as a single, unique pathway, whose activation depends on a variety of stimuli, such as growth factors (like insulin-like growth factors or epidermal growth factor), nutrients (like glucose), but also various cellular components (like ATP or O_2_). PI3Ks are lipid kinases. Upon receptor tyrosine kinase (RTK) activation by extracellular signals, PI3K is activated leading to the production of the second messenger phosphatidylinositol-3,4,5-triphosphate (PI3,4,5-P3), which in turn recruits a subset of signaling proteins to the membrane. Those proteins include the serine/threonine kinase Akt, which on its own modulates several cellular processes involved in cell survival and cell cycle progression. Akt regulates several downstream molecules, including mTOR, which, through its downstream effectors 4EBP1 and P70S6 kinase (S6K), is implicated in the initiation of ribosomal translation of mRNAs into proteins essential for cell growth, cell cycle progression, and cell metabolism. Besides being crucial for cell growth under normal conditions, the pathway is critical for survival under stress conditions. However, an aberrant activation of this signaling cascade can lead to different pathological states, including cancer [45].

It has been shown that the expression levels of *ECRG4* were significantly downregulated in nasopharyngeal carcinoma (NC) tissues compared with normal nasopharyngeal epithelial tissues and highly correlated with the prognosis of NC patients. Results of bioinformatic analysis showed that the expression of some target genes of the PI3K/Akt/mTOR signaling pathway were negatively correlated with *ECRG4* expression in NC cells, suggesting a potential contribution of augurin to the inhibition of the PI3K/Akt/mTOR signaling cascade. Indeed, in HONE-1 and SUNE-1 NC cells augurin overexpression decreased the phosphorylation levels of PI3K, Akt and mTOR and the expression levels of homologous recombinant repair (HRR) proteins [32]. It has been hypothesized that augurin inhibits the resistance of NC cells to radiotherapy and chemotherapy and their migratory ability through those mechanisms [32]. Overexpressed augurin inhibited Akt phosphorylation also in M2 squamous carcinoma head and neck (SCCHN) cancer cells [33], which may explain the augurin-mediated inhibition of SCCHN cells growth rate in vitro and in vivo.

**Fig. 3:**
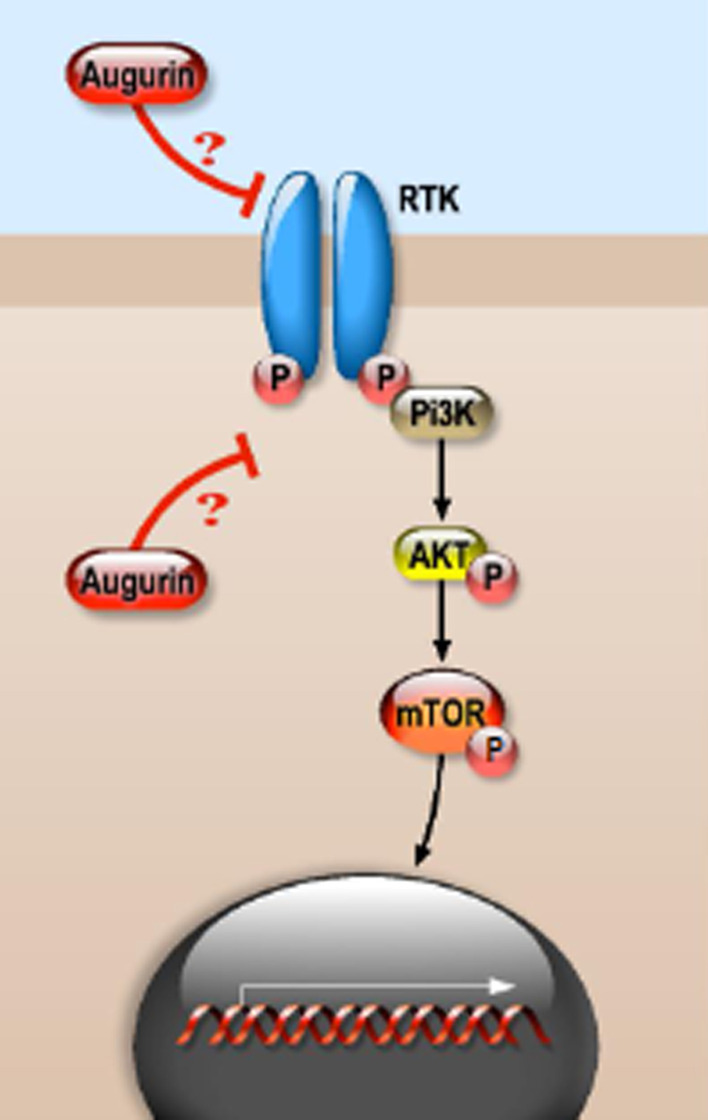
Augurin modulation of the PI3K/Akt/mTOR signaling pathway. A simplified schematic representation of the PI3K/Akt/mTOR signaling pathway is shown here. Activation of growth factor receptor protein tyrosine kinases (RTKs) results in autophosphorylation on tyrosine residues. PI3K is then recruited to the membrane by directly binding to phosphotyrosine consensus residues of RTKs and activated with subsequent production of the second messenger phosphatidylinositol-3,4,5-triphosphate (PI3,4,5-P3). PI3,4,5-P3 then recruits a subset of signaling proteins, including the Akt serine/threonine kinase, which, once phosphorylated and activated, can in turn activate the mTOR serine/threonine kinase. This kinase through its downstream effectors participates to the initiation of ribosomal translation of mRNAs into proteins, ultimately regulating cell growth and cell cycle progression. Augurin has been described to negatively regulate this signaling pathway [32, 33, 45], however it is still unknown at which level of the cascade it acts, the molecular mechanisms underlying the biological effects observed remaining to be defined

Those results then converge on a role of augurin as an inhibitor of PI3K/Akt/mTOR signaling (Fig. 3).

Another study has provided further insight into this regulatory mechanism. Indeed, it has been proposed that OGN inhibits epithelial-to-mesenchymal transition (EMT) via the PI3K/Akt/mTOR pathway. A decreased phosphorylation of Akt and mTOR has been reported in breast cancer cells overexpressing OGN together with a decreased expression of the EMT-associated molecular biomarkers N-cadherin and Snail1 [46]. Those results are consistent with the study cited in Sect.4.1, which shows that augurin inhibits the NF-κB pathway via its action on OGN [44]. It is thus possible to speculate that the increased OGN expression mediated by augurin might be one of the mechanisms through which augurin inhibits both the PI3K/Akt/mTOR and the NF-κB pathways.

### BC200 lncRNA/MMP-9 and -13 signaling pathway

Long non-coding RNA-BC2000 (LncRNA-BC2000), also known as BCYRN1, is a brain-specific small cytoplasmic RNA. LncRNAs are defined as transcripts of more of 200 nucleotides and containing little or no protein-coding potential. They are involved in the transcriptional and post-transcriptional regulation of various biological processes involved in tumor development [reviewed in [47]]. It has been shown that BC200 promotes the expression of matrix metalloproteinases 9 and 13 (MMP9 and MMP13) [48], two matrix metalloproteinases that catalyze the degradation of extracellular matrix (ECM) components responsible of the cleavage and release of biologically active molecules stored in the ECM, such as vascular endothelial growth factor A (VEGFA).

**Fig. 4:**
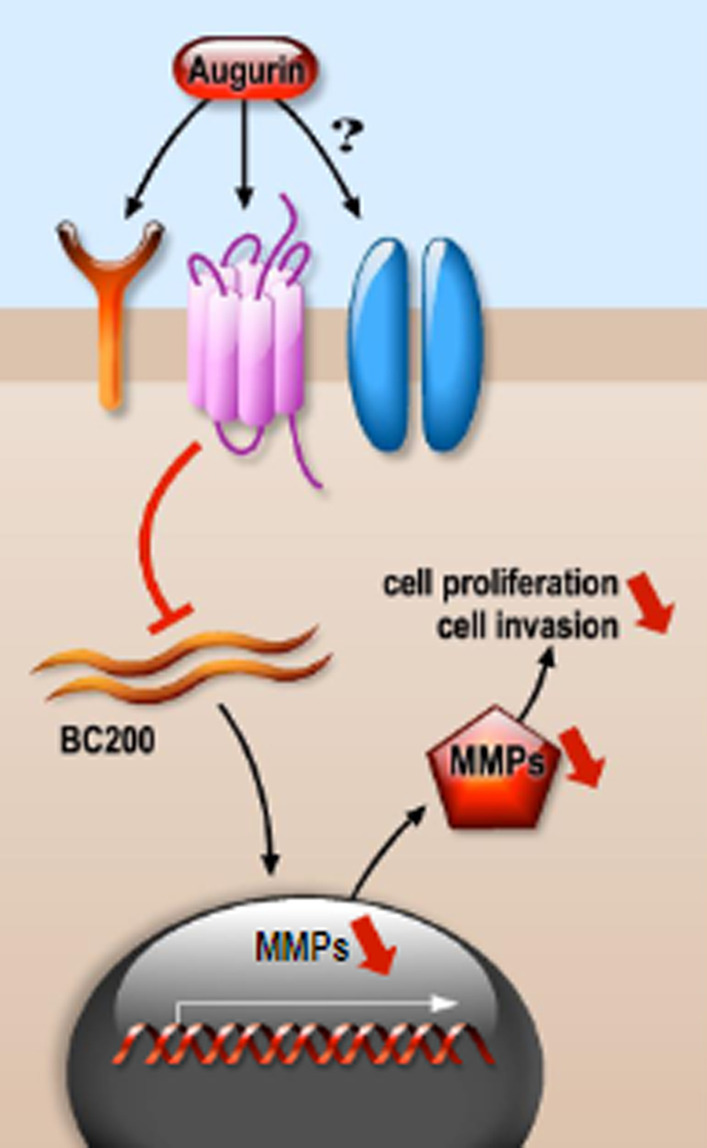
Augurin suppresses the BC200 lncRNA/MMP-9 and -13 signaling pathway. Forced expression of augurin down-regulates the expression of the BC200 lncRNA and matrix metalloproteinases MMP-9 and MMP-13, resulting in inhibition of cell migration/invasion and proliferation [48]. The molecular mechanisms underlying the augurin-mediated regulation of this signaling cascade, including the receptor through which augurin functions, remain largely unknown

Evidence provided by Huang and coworkers shows that augurin impacts the BC200 lncRNA/MMPs signaling pathway in the TCA8113 tongue carcinoma cell line [49]. A forced expression of *ECRG4* decreased the expression of BC200, which in turn decreased MMP13 and MMP9 levels (Fig. 4).

This resulted in an inhibition of cell proliferation and transmigration with a suppression of the malignant phenotype of TCA8113 cells. Restoration of BC200 lncRNA rescued augurin-mediated down-regulation of MMP-9 and -13 and cell proliferative and migratory properties [49]. However, how BC200 and *ECRG4,* which are both located on chromosome 2, regulate each other is still poorly explored and the regulation of this signaling cascade by augurin remains to be better elucidated.

### Apoptotic pathway

Apoptosis is a natural physiological phenomenon of programmed cell death, which is crucial for the maintenance of cellular homeostasis. We distinguish an extrinsic and an intrinsic apoptotic pathway. The extrinsic apoptotic pathway is mediated by cell surface death receptors that are members of the tumor necrosis factor (TNF) receptor superfamily such as the FAS receptor. Ligand binding to these death receptors induces a signaling cascade including the initiator caspases 8, which, once activated, will in turn activate other effector caspases such as caspase 3, ultimately leading to apoptosis. The intrinsic apoptotic pathway is mediated by the mitochondria and relies on the balance between anti- and pro- apoptotic proteins members of the Bcl-2 superfamily. The intrinsic pathway, when activated by cellular stress signals, leads to the up regulation of pro-apoptotic proteins such as BAD or BID. Those pro-apoptotic signals then induce the homo-oligomerization of BAK and BAX proteins and the permeabilization of mitochondria, which in turn release cytochrome c and lead to apoptosis via caspase 3. Activation of caspase 8 also results in the cleavage and activation of BID, which promotes BAX/BAK assembly, thus linking the intrinsic and extrinsic apoptosis pathways [reviewed in [50]].

**Fig. 5:**
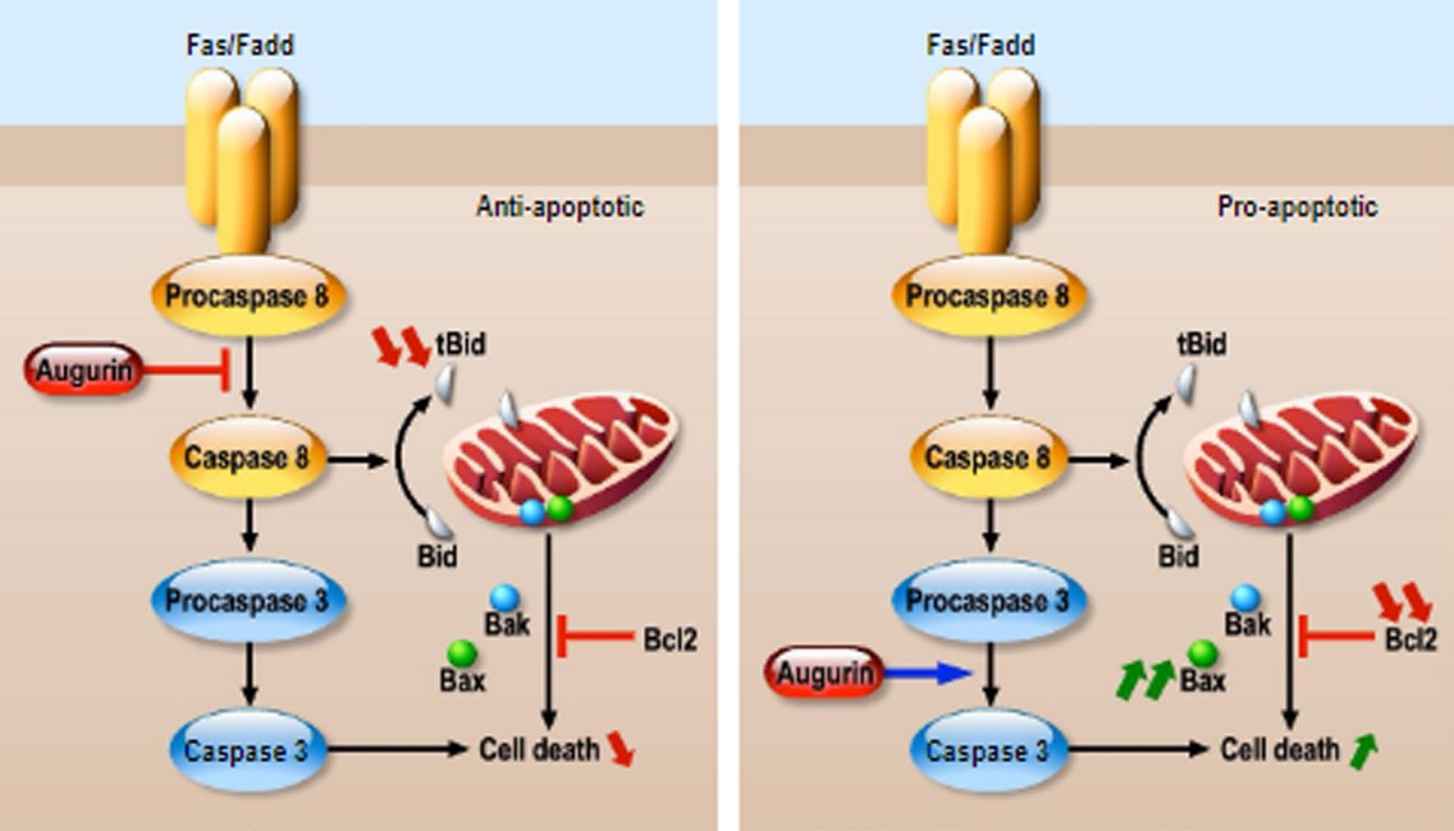
Augurin-mediated regulation of apoptotic signaling pathways. The activation of the FAS receptor induces the cleavage of procaspase 8 into caspase 8, which in turn induces the cleavage of procaspase 3 into caspase 3, finally leading to cell death. Caspase 8 can also promote the cleavage of BID, which is a pro-apoptotic molecule that induces Bax/Bak assembly, outer mitochondria membrane permeabilization and cytochrome c release, triggering the mitochondrial apoptotic pathway. Bcl2 is an anti-apoptotic factor which counteracts those actions. Augurin negatively regulates Fas-induced apoptosis in human T-leukemia cells by associating to procaspase 8 and preventing its cleavage to caspase 8 and consequently BID cleavage [50] (left panel). In other cellular models augurin seems to play a pro-apoptotic function increasing the expression levels of cleaved-PARP, cleaved-caspase-3 and Bax, while decreasing the expression levels of the anti-apoptotic factor Bcl2 (right panel), all those actions stimulating the mitochondrial apoptotic pathway [27, 32, 51]

Several studies have shown a role for augurin in regulating apoptotic pathways. One of them has provided evidence for augurin as a novel regulator of caspase-8-dependent apoptotic signal in human Jurkat and SUP T-13 T cells [51]. In this study, the authors first identified a VDAC2-like domain in augurin that can be associated with an apoptotic signal in the mitochondria and confirmed the augurin presence in those organelles by confocal microscopy. They then showed that when overexpressed in Fas-sensitive Jurkat cells, augurin inhibits the mitochondrial membrane permeability transition, leading to resistance to Fas-induced apoptosis, which suggests an inhibitory role for augurin towards this apoptotic mechanism. They also showed by immunoprecipitation that augurin is associated with procaspase-8 and suppresses the activation of caspase-8, thus leading to BID cleavage inhibition (Fig. 5).

Other studies have shown a pro-apoptotic role for augurin. Its overexpression activated caspase-3 and PARP, ultimately inducing apoptosis through the upregulation of the proapoptotic protein Bax and the downregulation of the antiapoptotic protein Bcl-2 (Fig. 5) in the laryngeal carcinoma Hep-2 and LSC-1 cell lines [30]. Similar observations were made in the hepatocellular carcinoma SMMC-7721 cell line [52]. Those results are consistent with another study performed in the M2 SCCHN cell line, where augurin was shown to increase the expression of Bax while decreasing the expression of Bcl-2 [33]. Overall, those findings revealed that augurin can trigger the mitochondrial apoptotic pathway through Bax/Bcl-2.

Augurin thus appears to be implicated in programmed cells death signaling pathways by playing a pro- or an anti- apoptotic role depending on the specific cell type.

### Wnt/β-catenin signaling pathway

**Fig. 6:**
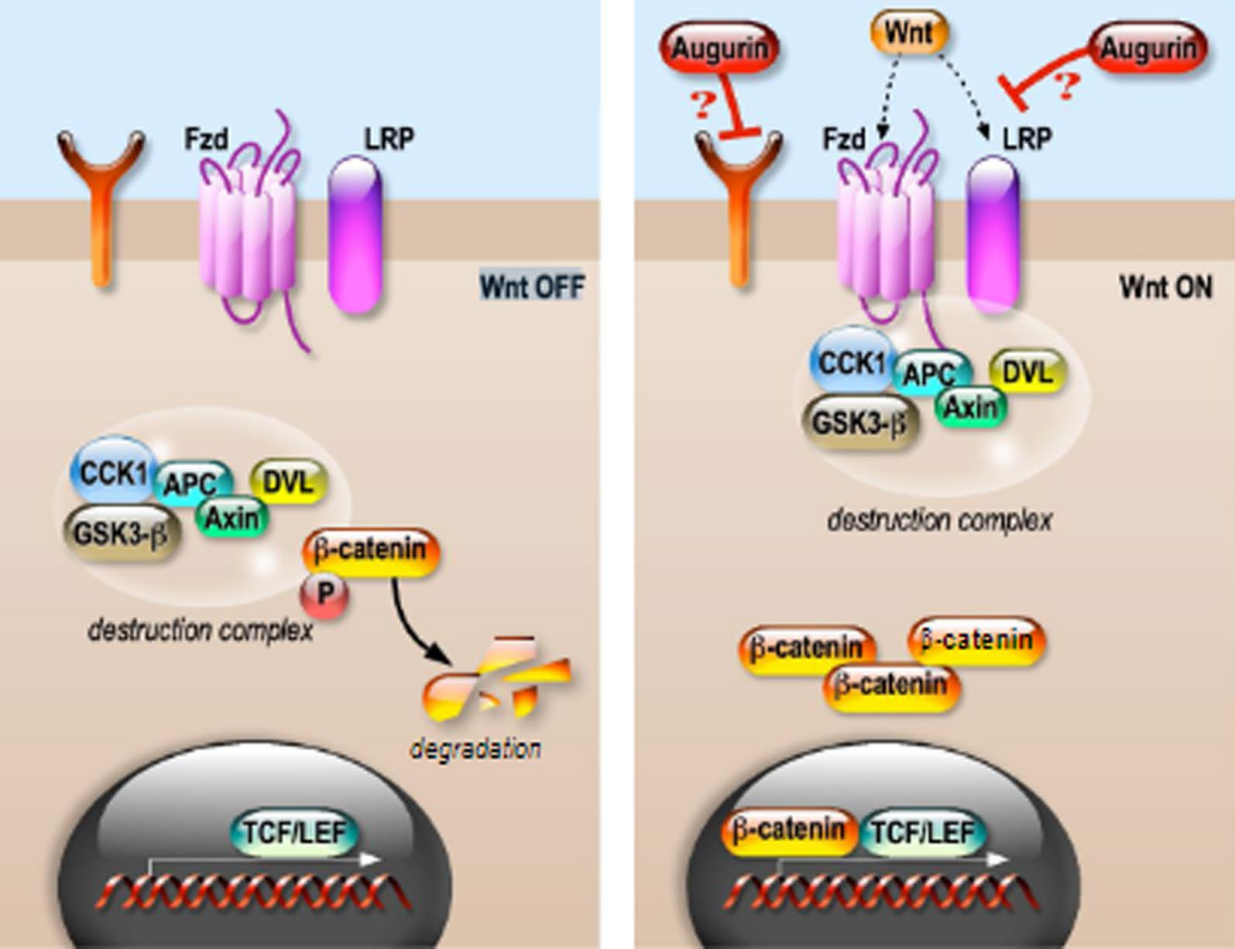
Augurin is an inhibitor of the canonical Wnt/ β-catenin signaling pathway. In the absence of WNT ligand, β-catenin is retained in a complex with axin, APC, CK1α and GSK-3β (destruction complex). Phosphorylation by CK1α and GSK-3β prepares β-catenin to undergo proteasomal degradation (left panel). Upon Wnt binding to Frizzled (Fzd) and LRP5/6, the destruction complex function is disrupted. The suppression of GSK-3β activity leads to β-catenin accumulation into the cytoplasm and translocation to the nucleus, where it interacts with the TCF/LEF transcription factors to activate the transcription of their target genes (right panel). Augurin specifically represses the canonical Wnt/β-catenin signaling pathway, however through which receptor it exerts its action and the precise mechanisms of Wnt signaling repression remain to be unveiled [37]

The Wnt signaling pathway plays a major role in development and is also essential for stem cell regulation and tissue homeostasis. Because of its crucial role in epithelial cell homeostasis, mutations in certain players of the Wnt pathway have been found in several cancers [53–55]. In the canonical Wnt/ β-catenin signaling pathway, the main player is β-catenin, whose stability is mediated by a destruction complex composed of Axin, that acts as a scaffolding protein interacting with β -catenin, the APC (Adenomatosis polyposis coli) tumor suppressor protein and two serine-threonine kinases, CK1 (casein kinase-1) and GSK3-β (glycogen synthase kinase 3-β) which is stabilized in its phosphorylated form [56]. In the absence of a Wnt ligand, the destruction complex mediates the degradation of β-catenin (Fig. 6). As soon as Wnt binds both the Frizzled (Fzd) receptor and coreceptors low-density lipoprotein receptor-related proteins 5 and 6 (LRP5/6) the destruction complex function is disrupted. This is due to Wnt causing the translocation of the negative Wnt regulator Axin and the destruction complex to the plasma membrane. This allows β-catenin to accumulate and localize to the nucleus and subsequently trigger a cellular response via gene regulation through the TCF/LEF (T-cell factor/lymphoid enhancing factor) transcription factors (Fig. 6).

We have first identified *Ecrg4* as a gene differentially expressed in the adrenal gland of *Kcnk3* mutant mice, a model of sexually dimorphic primary hyperaldosteronism [57]. In the rat [58] and mouse adrenal gland, augurin is specifically localized in the cortex zona glomerulosa, an area of active Wnt signaling [59]. Remarkably, *Ecrg4* is one of the most significantly repressed genes in a mouse model of Wnt signaling downregulation in the adrenal gland [60]. Overall, these data led us to the hypothesis that augurin might be implicated in the Wnt signaling pathway. Indeed, we have recently provided evidence that augurin is a novel physiological modulator of canonical Wnt signaling involved in osteoblast differentiation [37]. We have shown that osteoblasts from *Ecrg4* −/− mice displayed an accelerated differentiation compared to wild-type and upregulation of Wnt activation markers [37]. Furthermore, we could demonstrate that *Ecrg4* is itself a negative Wnt target gene, being markedly downregulated by the treatment of osteoblast-like mouse MC3T3-E1 cells with Wnt3a and RSPO1 [37]. In transient transfection assays in MC3T3-E1 cells, both mouse (mAug) and human augurin (hAug) significantly and specifically repressed activity of the Wnt-responsive TOPFLASH promoter upon stimulation with Wnt3a/RSPO1, the mutation of the putative furin cleavage site (R67A/K69A) in both mAug and hAug having no effects on the repression of TOPFLASH [37]. This evidence has been corroborated by data indicating that mAug also specifically repressed Wnt-stimulated TOPFLASH when expressed both as a secreted protein in CHO cells and as a recombinant protein in *E. coli*. As reported before, augurin was described to bind to LOX1 and other members of the scavenger receptor family [43]. We confirmed the binding of both mAug and hAug, expressed as alkaline phosphatase (AP) fusion proteins, to LOX1, while they did not bind to the Wnt and R-spondin coreceptors LRP5, LRP6 and LGR4. However, we could provide evidence that Wnt signaling inhibition by augurin is not mediated by its binding to scavenger receptors.

As reported above, augurin can either activate or repress NF-κB signaling depending on the cell type [22, 23, 43, 44]. Both positive and negative crosstalk between the NF-κB and Wnt signaling pathways has been reported [61], the possibility existing that the effects of augurin on Wnt signaling in osteoblastic cells may be mediated by NF-κB activity modulation. However, the activity of a transfected NF-κB reporter by treatment of MC3T3-E1 cells with recombinant augurin was not modulated [37]. Those results suggest that in MC3T3-E1 cells augurin represses Wnt-stimulated transcription independently from NF-κB. However, further experiments are needed to elucidate the mechanisms of Wnt signaling repression by augurin in osteoblastic cells.

In line with those findings, a recent study has described an inhibitory action for augurin on the Akt/GSK3β / β-catenin pathway in NC cells [62]. Augurin overexpression in NC cells resulted in the downregulation of the expression levels of β-catenin and its downstream targets cyclin D1 and Akt and phosphorylation of Akt (on Tyr308/450 and Ser473) and of GSK3-β (on Ser9). Opposite results were observed in augurin-depleted NC cells. Those findings correlated with reduced mRNA expression levels of the downstream Wnt targets *Cyclin E*, *Cyclin D1*, *c-Myc*, *c-Jun* and *Axin*2 in augurin-overexpressing NC cells.

Considering the expression pattern of *ECRG4*/augurin in several tissues under physiological conditions and its alterations in disease, the novel role of augurin as a canonical Wnt signaling inhibitor might underpin many of its described effects on cancer cell proliferation and tumor growth [23, 25], cell senescence [16], stem cell renewal [3, 17], and inflammation/response to tissue injury [20, 21].

## Concluding remarks

Augurin has emerged over the last few years as an intriguing protein, whose physiological roles include functioning as a tumor suppressor protein, a neuropeptide, a sentinel molecule, a cytokine-chemokine, all those roles converging to support its implication in cancer, injury, inflammation, infection, stem cell renewal and tissue homeostasis. An interesting aspect concerning augurin biology is represented by its activity resembling cytokines-chemokines-growth factors, all molecules that need proteolytic processing for activity. Further, augurin processing, which is cell type-dependent, produces multiple peptides, which, like several neuropeptide precursors, can play synergistic or even opposing biologically activities, leading to antagonistic responses (e.g. pro- or anti- apoptotic, pro- or anti-inflammatory).

Here we have tried to provide a picture as complete as possible of the signaling pathways that augurin regulates to exert its functions. However, it clearly emerges from this picture that most of the studies carried out so far lack an in-depth characterization of the molecular mechanisms underlying the biological actions of augurin and derived peptides and of the modulation of the expression and/or activity of augurin downstream signaling molecules. The understanding of those mechanisms is crucial to provide the essential background for the development of specific agonists and antagonists able to modulate augurin-dependent signaling cascades. Considering the involvement of those pathways in known human pathologies, like cancer, inflammatory and immunological diseases, neurological disorders, the definition of the molecular mechanisms through which augurin contributes to their modulation is of utmost importance.

To this end, the characterization of the precise nature of augurin-derived peptides and the identification of the receptor(s) on the cell surface conveying augurin signaling to downstream effectors are crucial and represent the critical prerequisite for the development of agonists and antagonists for this protein. Indeed, their secreted nature and the potential to be manipulated pharmacologically make augurin and its peptides attractive targets for diagnostic development and discovery of new therapeutic agents for human diseases resulting from the deregulation of the signaling pathways where augurin is implicated.

## Data Availability

Not applicable.

## Abbreviations

AP: Alkaline phosphatase
APC: Adenomatosis polyposis coli
CNS: Central nervous system
CK1: Casein kinase-1
CRH: Corticotropin-releasing hormone
EMT: Epithelial-to-mesenchymal transition
ECRG4: Esophageal cancer-related gene 4
ECM: Extracellular matrix
Fzd: Frizzled
GSK3-β: Glycogen synthase kinase 3-β
HRR: Homologous recombinant repair
hAug: Human augurin
HPA: Hypothalamo-pituitary-adrenal
LncRNA: Long non-coding RNA
LRP: Low-density lipoprotein receptor-related protein
mTOR: Mammalian target of rapamycin
mAug: Mouse augurin
MMP: Matrix metalloproteinase
NC: Nasopharyngeal carcinoma
NFIC: Nuclear factor 1 C-type
NF-κB: Nuclear factor kappa-light-chain-enhancer of activated B cells
NPC: Neural progenitor cell
NSC: Neural stem cell
OPCs: Oligodendrocyte precursor cell
OGN: Osteoglycin
PI3K: Phosphatidylinositol-3-kinase
PI3,4,5-P3: Phosphatidylinositol-3,4,5-triphosphate
RTK: Receptor tyrosine kinase
SCCHN: Squamous cell carcinoma of head and neck
TCF/LEF: T-cell factor/lymphoid enhancing factor
TLR: Toll like receptor
TNF: Tumor necrosis factor
VEGFA: Vascular endothelial growth factor A

## References

[1] Mirabeau O, Perlas E, Severini C, Audero E, Gascuel O, Possenti R (2007). Identification of novel peptide hormones in the human proteome by hidden Markov model screening. Genome Res.

[2] Su T, Liu H, Lu S (1998). Cloning and identification of cDNA fragments related to human esophageal cancer. Zhonghua Zhong Liu Za Zhi.

[3] Gonzalez AM, Podvin S, Lin SY, Miller MC, Botfield H, Leadbeater WE (2011). Ecrg4 expression and its product augurin in the choroid plexus: impact on fetal brain development, cerebrospinal fluid homeostasis and neuroprogenitor cell response to CNS injury. Fluids Barriers CNS.

[4] Dang X, Podvin S, Coimbra R, Eliceiri B, Baird A (2012). Cell specific processing and release of the hormone-like precursor and candidate tumor suppressor gene product, Ecrg4. Cell Tissue Res.

[5] Huh YH, Ryu JH, Shin S, Lee DU, Yang S, Oh KS (2009). Esophageal cancer related gene 4 (ECRG4) is a marker of articular chondrocyte differentiation and cartilage destruction. Gene.

[6] Dowell JA, Johnson JA, Li L (2009). Identification of astrocyte secreted proteins with a combination of shotgun proteomics and bioinformatics. J Proteome Res.

[7] Tachibana N, Fujimaki T, Nakamura S, Funaki T, Murakami A (2002). Gene expression profile study of corneal endothelium. Juntendo Med J.

[8] Horiuchi T, Mitoma H, Harashima S, Tsukamoto H, Shimoda T (2010). Transmembrane TNF-alpha: structure, function and interaction with anti-TNF agents. Rheumatol Oxf Engl.

[9] Ozawa A, Lick AN, Lindberg I (2011). Processing of proaugurin is required to suppress proliferation of tumor cell lines. Mol Endocrinol.

[10] Feige Jj, Baird A. Crinopexy: extracellular regulation of growth factor action. Kidney Int Suppl. 1995;49. 7674586

[11] Matsuzaki J, Torigoe T, Hirohashi Y, Tamura Y, Asanuma H, Nakazawa E (2013). Expression of ECRG4 is associated with lower proliferative potential of esophageal cancer cells. Pathol Int.

[12] Bi MX, Han WD, Lu SX (2001). Using lab on-line to clone and identify the esophageal cancer related gene 4. Sheng Wu Hua Xue Yu Sheng Wu Wu Li Xue Bao Acta Biochim Biophys Sin.

[13] Kao S, Shaterian A, Cauvi DM, Dang X, Chun HB, De Maio A (2015). Pulmonary preconditioning, injury, and inflammation modulate expression of the candidate tumor suppressor gene ECRG4 in lung. Exp Lung Res.

[14] Su AI, Wiltshire T, Batalov S, Lapp H, Ching KA, Block D (2004). A gene atlas of the mouse and human protein-encoding transcriptomes. Proc Natl Acad Sci.

[15] Roberton A, Gonzalez AM, Stopa E, Leadbeater W, Coimbra R, Johanson C, et al. Immunohistochemical evidence that Argillin, the product of the ECRG4 gene, encodes a novel neuroendocrine peptide. Endocr Abstr. 2009;19.

[16] Kujuro Y, Suzuki N, Kondo T (2010). Esophageal cancer-related gene 4 is a secreted inducer of cell senescence expressed by aged CNS precursor cells. Proc Natl Acad Sci.

[17] Nakatani Y, Kiyonari H, Kondo T (2019). Ecrg4 deficiency extends the replicative capacity of neural stem cells in a Foxg1-dependent manner. Dev Camb Engl.

[18] Podvin S, Dang X, Meads M, Kurabi A, Costantini T, Eliceiri BP (2015). Esophageal cancer-related gene-4 (ECRG4) interactions with the innate immunity receptor complex. Inflamm Res Off J Eur Histamine Res Soc Al.

[19] Podvin S, Gonzalez AM, Miller MC, Dang X, Botfield H, Donahue JE (2011). Esophageal cancer related gene-4 is a choroid plexus-derived injury response gene: evidence for a biphasic response in early and late brain injury. PLoS ONE.

[20] Kurabi A, Pak K, Dang X, Coimbra R, Eliceiri BP, Ryan AF (2013). Ecrg4 attenuates the inflammatory proliferative response of mucosal epithelial cells to infection. PLoS ONE.

[21] Shaterian A, Kao S, Cauvi D, DeMaio A, Chun H, Costantini T (2013). Identification of Ecrg4 and its cytokine-like protein product in lung: differential down regulation after exploratory laparotomy. J Surg Res.

[22] Baird A, Coimbra R, Dang X, Lopez N, Lee J, Krzyzaniak M (2012). Cell surface localization and release of the candidate tumor suppressor Ecrg4 from polymorphonuclear cells and monocytes activate macrophages. J Leukoc Biol.

[23] Li LW, Yu XY, Yang Y, Zhang CP, Guo LP, Lu SH (2009). Expression of esophageal cancer related gene 4 (ECRG4), a novel tumor suppressor gene, in esophageal cancer and its inhibitory effect on the tumor growth in vitro and in vivo. Int J Cancer.

[24] Yue CM, Deng DJ, Bi MX, Guo LP, Lu SH (2003). Expression of ECRG4, a novel esophageal cancer-related gene, downregulated by CpG island hypermethylation in human esophageal squamous cell carcinoma. World J Gastroenterol.

[25] Cai Z, Liang P, Xuan J, Wan J, Guo H (2016). ECRG4 as a novel tumor suppressor gene inhibits colorectal cancer cell growth in vitro and in vivo. Tumor Biol.

[26] Li W, Liu X, Zhang B, Qi D, Zhang L, Jin Y (2010). Overexpression of candidate tumor suppressor ECRG4 inhibits glioma proliferation and invasion. J Exp Clin Cancer Res CR.

[27] Götze S, Feldhaus V, Traska T, Wolter M, Reifenberger G, Tannapfel A (2009). ECRG4 is a candidate tumor suppressor gene frequently hypermethylated in colorectal carcinoma and glioma. BMC Cancer.

[28] Sabatier R, Finetti P, Adelaide J, Guille A, Borg JP, Chaffanet M (2011). Down-regulation of ECRG4, a candidate tumor suppressor gene, in human breast cancer. PLoS ONE.

[29] Vanaja DK, Ehrich M, Van den Boom D, Cheville JC, Karnes RJ, Tindall DJ (2009). Hypermethylation of genes for diagnosis and risk stratification of prostate cancer. Cancer Invest.

[30] Jia J, Dai S, Sun X, Sang Y, Xu Z, Zhang J, Cui X, Song J, Guo X (2015). A preliminary study of the effect of ECRG4 overexpression on the proliferation and apoptosis of human laryngeal cancer cells and the underlying mechanisms. Mol Med Rep.

[31] Lee J, Dang X, Borboa A, Coimbra R, Baird A, Eliceiri BP (2015). Thrombin-processed Ecrg4 recruits myeloid cells and induces antitumorigenic inflammation. Neuro-Oncology.

[32] Xie Z, Li W, Ai J, Xie J, Zhang X (2022). C2orf40 inhibits metastasis and regulates chemo-resistance and radio-resistance of nasopharyngeal carcinoma cells by influencing cell cycle and activating the PI3K/AKT/mTOR signaling pathway. J Transl Med.

[33] Xu T, Xiao D, Zhang X (2013). ECRG4 inhibits growth and invasiveness of squamous cell carcinoma of the head and neck in vitro and in vivo. Oncol Lett.

[34] Baird A, Lee J, Podvin S, Kurabi A, Dang X, Coimbra R (2014). Esophageal cancer-related gene 4 at the interface of injury, inflammation, infection, and malignancy. Gastrointest Cancer Targets Ther.

[35] Qin X, Zhang P (2019). ECRG4: a new potential target in precision medicine. Front Med.

[36] Tadross J, Patterson M, Suzuki K, Beale K, Boughton C, Smith K (2010). Augurin stimulates the hypothalamo-pituitary-adrenal axis via the release of corticotrophin-releasing factor in rats. Br J Pharmacol avr.

[37] Ruggiero C, Durand N, Jarjat M, Barhanin J, Ghirardello EJ, Dack MRG (2022). The secreted protein augurin is a novel modulator of canonical Wnt signalling involved in osteoblast differentiation. Clin Transl Discov.

[38] Liu T, Zhang L, Joo D, Sun SC (2017). NF-κB signaling in inflammation. Signal Transduct Target Ther.

[39] Oeckinghaus A, Ghosh S (2009). The NF-kappaB family of transcription factors and its regulation. Cold Spring Harb Perspect Biol.

[40] Moynagh PN (2005). The NF-κB pathway. J Cell Sci.

[41] Podvin S, Dang X, Meads M, Kurabi A, Costantini T, Eliceiri BP (2015). Esophageal cancer related gene-4 (ECRG4) interactions with the innate immunity receptor complex. Inflamm Res Off J Eur Histamine Res Soc Al.

[42] Dang X, Coimbra R, Mao L, Podvin S, Li X, Yu H (2019). Open reading frame mining identifies a TLR4 binding domain in the primary sequence of ECRG4. Cell Mol Life Sci.

[43] Moriguchi T, Takeda S, Iwashita S, Enomoto K, Sawamura T, Koshimizu U (2018). Ecrg4 peptide is the ligand of multiple scavenger receptors. Sci Rep.

[44] Liang X, Gao J, Wang Q, Hou S, Wu C (2020). ECRG4 represses cell proliferation and invasiveness via NFIC/OGN/NF-κB signaling pathway in bladder cancer. Front Genet.

[45] Luo Q, Du R, Liu W, Huang G, Dong Z, Li X (2022). PI3K/Akt/mTOR signaling pathway: role in esophageal squamous cell carcinoma, regulatory mechanisms and opportunities for targeted therapy. Front Oncol.

[46] Xu T, Zhang R, Dong M, Zhang Z, Li H, Zhan C (2019). Osteoglycin (OGN) inhibits cell proliferation and invasiveness in breast cancer via PI3K/Akt/mTOR signaling pathway. OncoTargets Ther.

[47] Ghafouri-Fard S, Dashti S, Hussen BM, Farsi M, Taheri M (2021). BCYRN1: an oncogenic lncRNA in diverse cancers. Pathol Res Pract.

[48] Hu T, Lu YR. BCYRN1, a c-MYC-activated long non-coding RNA, regulates cell metastasis of non-small-cell lung cancer. Cancer Cell Int. 1 avr 2015;15:36. 10.1186/s12935-015-0183-3 PMC4392634 25866480

[49] Huang W, Zhou R, Mao L, Deng C, Dang X (2019). Esophageal cancer related gene-4 inhibits the migration and proliferation of oral squamous cell carcinoma through BC200 lncRNA/MMP-9 and -13 signaling pathway. Cell Signal.

[50] Khan KH, Blanco-Codesido M, Molife LR (2014). Cancer therapeutics: targeting the apoptotic pathway. Crit Rev Oncol Hematol.

[51] Matsuzaki J, Torigoe T, Hirohashi Y, Kamiguchi K, Tamura Y, Tsukahara T (2012). ECRG4 is a negative regulator of caspase-8-mediated apoptosis in human T-leukemia cells. Carcinogenesis.

[52] Ge S, Xu Y, Wang H, Sun Y, Tian X, Cao Z (2017). Downregulation of esophageal cancer-related gene 4 promotes proliferation and migration of hepatocellular carcinoma. Oncol Lett sept.

[53] Morin PJ, Sparks AB, Korinek V, Barker N, Clevers H, Vogelstein B (1997). Activation of β-catenin-Tcf signaling in colon cancer by mutations in β-catenin or APC. Science.

[54] Kinzler KW, Nilbert MC, Vogelstein B, Bryan TM, Levy DB, Smith KJ (1991). Identification of a gene located at chromosome 5q21 that is mutated in colorectal cancers. Science.

[55] Nishisho I, Nakamura Y, Miyoshi Y, Miki Y, Ando H, Horii A (1991). Mutations of chromosome 5q21 genes in FAP and colorectal cancer patients. Science.

[56] Stamos JL, Weis WI (2013). The β-catenin destruction complex. Cold Spring Harb Perspect Biol.

[57] El Wakil A, Bandulik S, Guy N, Bendahhou S, Zennaro MC, Niehrs C (2012). *Dkk3* is a component of the genetic circuitry regulating aldosterone biosynthesis in the adrenal cortex. Hum Mol Genet.

[58] Porzionato A, Rucinski M, Macchi V, Sarasin G, Malendowicz LK, De Caro R (2015). ECRG4 expression in normal rat tissues: expression study and literature review. Eur J Histochem EJH.

[59] Pignatti E, Leng S, Carlone DL, Breault DT (2017). Regulation of zonation and homeostasis in the adrenal cortex. Mol Cell Endocrinol.

[60] Drelon C, Berthon A, Sahut-Barnola I, Mathieu M, Dumontet T, Rodriguez S (2016). PKA inhibits WNT signalling in adrenal cortex zonation and prevents malignant tumour development. Nat Commun.

[61] Ma B, Hottiger MO (2016). Crosstalk between Wnt/β-catenin and NF-κB signaling pathway during inflammation. Front Immunol.

[62] Yang Z, Ye X, Zhang Y, Huang Y, Chen J, Zeng Y (2022). *ECRG4* acts as a tumor suppressor in nasopharyngeal carcinoma by suppressing the AKT/GSK3β/β-catenin signaling pathway. Cytotechnology.

